# Backbone‐Length‐Optimized Inhibitors Deliver Long‐Retention Selectivity in Area‐Selective ALD of VO_2_

**DOI:** 10.1002/advs.75705

**Published:** 2026-05-15

**Authors:** Hae Lin Yang, Eun Chong Cho, Minchan Kim, Hye In Park, Ga‐young Lee, Seunghwan Lee, Beomseok Kim, Changhwa Jung, Youngkwon Kim, Jin‐Seong Park

**Affiliations:** ^1^ Division of Materials Science and Engineering Hanyang University Seoul Republic of Korea; ^2^ Thin Film Materials Research Center Research Institute of Chemical Technology (KRICT) Daejeon Republic of Korea; ^3^ Department of Semiconductor Engineering Hanyang University Seoul Republic of Korea; ^4^ Semiconductor Research and Development Center Samsung Electronics Company Hwaseong‐si Gyeonggi‐do Republic of Korea

**Keywords:** adsorption behavior, area‐selective atomic‐layer deposition, small‐molecule inhibitor, steric hindrance, vanadium oxide

## Abstract

Area‐selective atomic‐layer deposition (AS‐ALD) relies on surface inhibitors; however, their molecular design remains empirical owing to the lack of a unified framework linking the molecular structure to inhibition performance. In this study, we systematically investigate the influence of the carbon‐backbone length of trimethoxyphenyl(alkyl)silane small‐molecule inhibitors (SMIs) on adsorption behavior and selectivity in the AS‐ALD of VO_2_. We use density functional theory to show that SMI adsorption on hydroxylated SiO_2_ proceeds through a multistep pathway involving physisorption and single‐ and double‐bonded chemisorption. Longer backbones enhance physisorption via stronger dispersion interactions; however, they impose rapidly increasing kinetic barriers to form a stable double‐bonded configuration owing to severe steric and conformational constraints. Random sequential adsorption simulations show that the achievable surface coverage is dictated by a tradeoff between the molecular packing density and steric exclusion, resulting in a nonmonotonic dependence on the backbone length. These coupled chemical and geometric effects define the optimal intermediate backbone‐length range. This prediction is validated by VO_2_ AS‐ALD experiments, in which only intermediate‐length SMIs maintain high selectivity (> 90%) over extended cycling. This study establishes a predictive and chemically and geometrically grounded design principle for molecular inhibitors in AS‐ALD, and broadly for area‐selective atomic‐layer processing.

## Graphical abstract

Area‐selective VO_2_ ALD is found to depend critically on inhibitor backbone length, which governs physisorption, chemisorption stability, and packing efficiency in a coupled manner. An intermediate backbone‐length achieves the best long‐retention selectivity, establishing a chemically and geometrically grounded design principle for small‐molecule inhibitors.

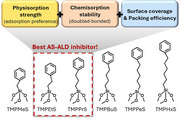

## 1 Introduction

Area‐selective atomic layer deposition (AS‐ALD) is a promising fabrication technique for advanced semiconductor devices because it enables film growth exclusively on the target surface, while suppressing nucleation on nongrowth surfaces [1, 2]. As device architectures evolve toward higher integration densities and increasingly complicated high‐aspect‐ratio geometries, the fraction and complexity of nongrowth regions inevitably increase. Under such conditions, even minor unintended nucleation on nongrowth surfaces can accumulate over repeated ALD cycles and eventually degrade the dimensional control and device performance [3, 4]. Therefore, the performance of AS‐ALD is increasingly defined by both the initial selectivity cumulative stability of the selectivity, that is, the ability to continuously suppress nucleation during extended process cycling [5, 6]. Achieving this stability requires the reliable control of the surface reactivity on nongrowth regions, and surface inhibitors have been widely adopted as an effective approach [7, 8, 9].

Surface inhibitors used in AS‐ALD are typically categorized into self‐assembled monolayers (SAMs) and small‐molecule inhibitors (SMIs). SAMs have shown strong inhibition performance in early studies; however, their practical use is limited by their long formation times, large molecular sizes, and reduced compatibility with fully vapor‐phase process integration [10, 11]. In addition, uniform monolayer formation has become an increasingly challenging task for confined and structurally complex device geometries [12]. These considerations have motivated growing interest in SMIs as a more practical alternative for AS‐ALD applications [13].

SMIs suppress nucleation on nongrowth surfaces, primarily through two mechanisms: chemical passivation and physical blocking [14, 15]. Chemical passivation occurs when the SMI head group competitively occupies reactive‐surface sites prior to precursor adsorption, thereby reducing the number of available reaction sites and inducing a nucleation delay [16]. In contrast, physical blocking originates from the steric hindrance imposed by adsorbed SMI molecules. Notably, effective inhibition can be achieved even when only a partial spatial overlap exists between the inhibitor‐binding and precursor‐adsorption sites [15]. This implies that precursor access can be hindered, even when some reactive sites remain exposed. Therefore, unlike for SAMs, effective inhibition by SMIs does not require the formation of densely packed and fully continuous monolayers [17].

The concepts of chemical passivation and physical blocking provide a useful description; however, these SMI structure–function relationships are sometimes simplified by assigning the head group to chemical passivation and blocking group to post‐adsorption steric hindrance [18, 19, 20, 21]. Previous studies on surface inhibitors, including SAM‐based systems and SMIs, have largely interpreted inhibition behavior in terms of static surface coverage and steric exclusion under limited or single‐cycle conditions [12, 22, 23]. While these approaches have provided valuable insights into initial nucleation delay and blocking efficiency, they do not fully capture how inhibition performance evolves during repeated ALD cycling. As a result, the role of molecular structure—particularly the backbone length—in governing adsorption dynamics, intermolecular interactions, and the stability of inhibition remains insufficiently understood [20, 21, 24].

Such conventional interpretations implicitly treat SMI adsorption as a static process. However, in practical AS‐ALD, SMIs are delivered in the vapor phase and adsorb sequentially, where the orientation and bonding configuration of the adsorbed molecules can influence subsequent adsorption events [25, 26]. From this dynamic perspective, the blocking group may limit precursor access after adsorption and influence adsorption pathways and bonding‐state formation during adsorption. Furthermore, the molecular size and backbone structure can modulate surface‐dispersion interactions [27, 28], conformational freedom [29, 30], and intermolecular interactions. This may potentially lead to distinct changes in the adsorption energy and chemisorption configurations, even for SMIs sharing the same head group. These considerations suggest that the blocking group should be treated as an active design parameter governing adsorption behavior and bonding‐state evolution, rather than merely a geometric steric barrier.

This study systematically investigates the influence of the carbon‐backbone length of trimethoxyphenyl(alkyl)silane SMIs on the adsorption and inhibition performance in the AS‐ALD of vanadium dioxide (VO_2_). We synthesize a series of six SMIs with identical trimethoxysilane head and phenyl groups while varying only the alkyl backbone length (‐methyl (TMPMeS), ‐ethyl (TMPEtS), ‐propyl (TMPPrS), ‐butyl (TMPBuS), ‐pentyl (TMPPeS), and ‐hexyl (TMPHxS)). Their thermal suitability for vapor‐phase processing is verified and their adsorption behavior on SiO_2_ is analyzed using a stepwise model encompassing physisorption and successive chemisorption states. Density functional theory (DFT) calculations and random sequential adsorption (RSA) simulations are combined with experimental surface characterization and AS‐ALD selectivity tests to correlate the molecular structure with adsorption energetics, surface coverage, and selectivity retention over repeated ALD cycles. Through this integrated approach, we reinterpret backbone‐dependent “blocking” effects as a coupled outcome of adsorption processes and bonding‐state formation, providing molecular design guidelines for the stable, high‐selectivity AS‐ALD of VO_2_.

## 2 Results and Discussion

**FIGURE 1:**
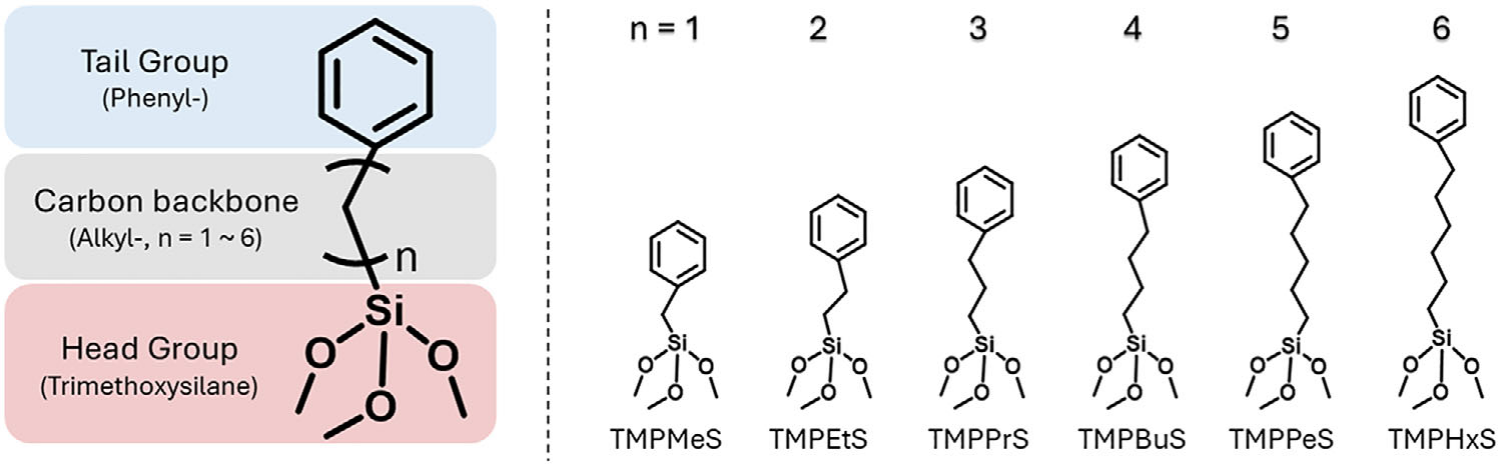
Molecular structures of trimethoxysilane‐based SMIs with varied alkyl backbone lengths. All molecules comprise an identical phenyl tail group and a trimethoxysilane head group, while the carbon backbone length (*n* = 1–6) is incrementally increased to isolate the effect of the backbone carbon number on molecular structure and surface interactions. The corresponding SMI series includes TMPMeS, TMPEtS, TMPPrS, TMPBuS, TMPPeS, and TMPHxS.

**SCHEME 1:**
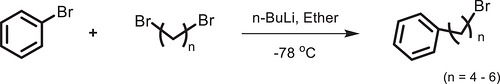
Synthetic process of 1‐bromo‐n‐phenylalkane.

**SCHEME 2:**
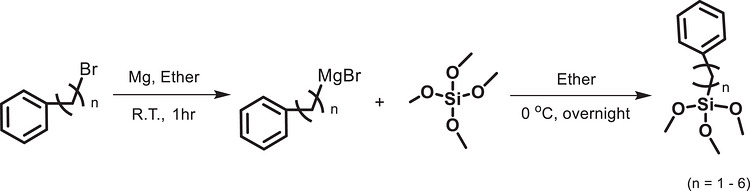
Synthesis of TMP(alkyl)S as SMI for AS‐ALD process.

As shown in Figure 1, we designed a series of six SMIs by dividing the molecular structure into a head group, carbon backbone, and tail group and systematically varying only the length of the carbon backbone between the head and tail groups. The synthetic routes for the precursor molecules and the corresponding SMI structures are described in Schemes 1 and 2, respectively. This molecular design enables the systematic investigation of the influence of the carbon‐backbone length on the inhibition behavior in the AS‐ALD process. In many previous studies, the SMI structures have been interpreted using a binary framework comprising a reactive group that chemically binds to the surface and blocking group that suppresses precursor adsorption. While this approach provides an intuitive description of inhibition mechanisms, it inherently treats the blocking group as a single structural entity. This limits the ability to distinguish the individual roles of the internal components of the blocking group during the adsorption and inhibition processes. In particular, the blocking group generally consists of a tail group exposed at the surface after adsorption and carbon backbone that forms the molecular scaffold, which can contribute to the SMI behavior in fundamentally different ways.

The tail group mainly contributes to chemical passivation by reducing surface reactivity, while the carbon backbone determines the geometric configuration of the adsorbed molecule, shaping its steric footprint on the surface. Therefore, in this study, the region conventionally referred to as the blocking group was separated into the tail group and carbon backbone for independent analysis. The tail group was fixed as a phenyl ring to provide a rigid and bulky moiety [31, 32, 33], while the head group was maintained as an alkoxysilane [21, 34]. In contrast, only the length of the carbon backbone was systematically varied from *n* = 1 to *n* = 6. The alkyl backbone can modulate the molecular orientation and rearrangement on the surface, thereby altering the packing density and local surface energy [35, 36, 37]. This can affect the precursor access and reaction behavior during subsequent ALD cycles. Such effects are particularly relevant for vapor‐phase SMI processes that rely on short exposure times; as a result, the backbone‐length‐dependent behavior may differ from trends commonly reported for solution‐processed SAMs [13, 38, 39].

**FIGURE 2:**
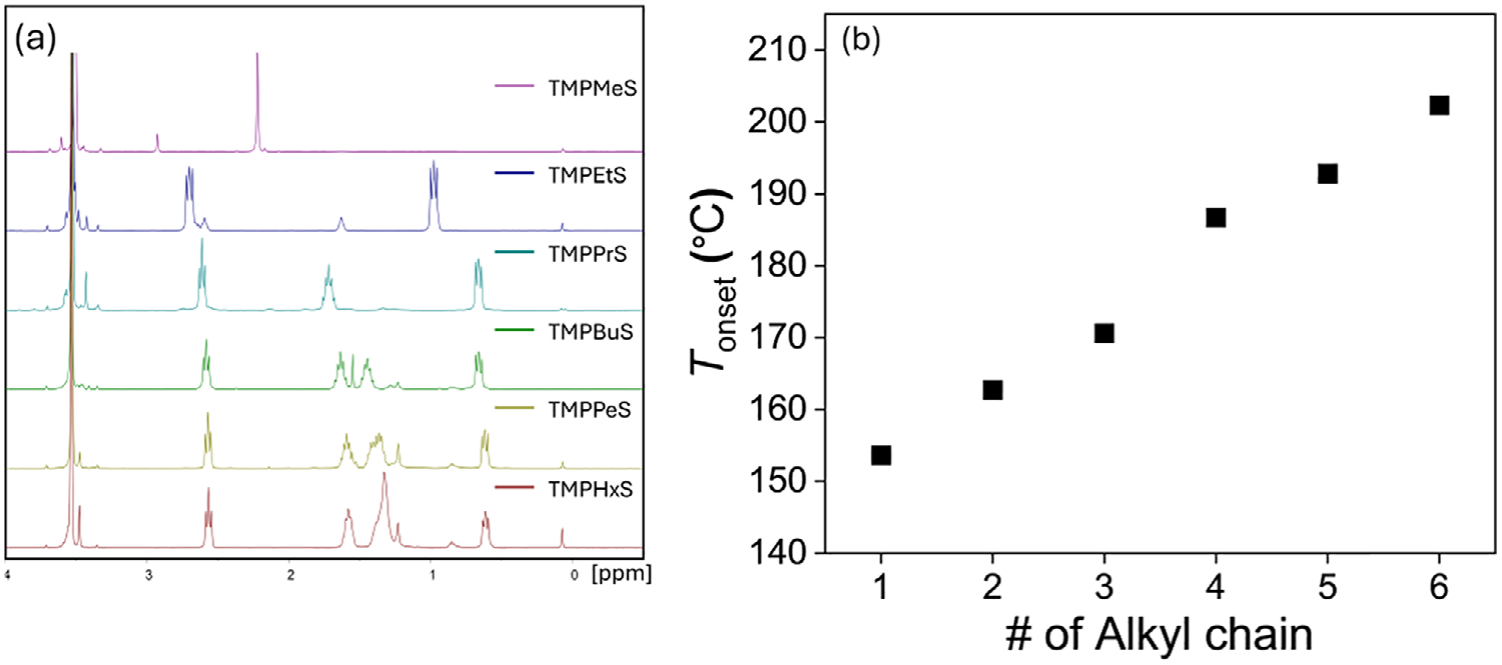
(a) 1H NMR spectra of trimethoxysilane‐based SMIs with different alkyl backbone lengths (TMPMeS, TMPEtS, TMPPrS, TMPBuS, TMPPeS, and TMPHxS). (b) Onset temperature of evaporation (Tonset) of the SMIs as a function of the alkyl chain number.

Next, the fundamental material properties were analyzed to verify that the molecular design presented in Figure 1 was successfully realized through synthesis and to evaluate the applicability of the synthesized SMIs to AS‐ALD processes. Figure S1 shows the full ^1^H nuclear magnetic resonance (NMR) spectra of the SMI series with alkyl backbone lengths ranging from 1 to 6. To compare the backbone‐length‐dependent signal evolution more clearly, only the alkyl‐chain region (0–4.0 ppm) was extracted and compiled, as shown in Figure 2a. For all SMIs, characteristic methoxy proton resonances originating from the trimethoxysilane head group were consistently observed at approximately 3.51–3.54 ppm, serving as a common reference peak across the entire series. As the carbon backbone length increased from methyl to hexyl, additional alkyl‐related methylene and methyl proton signals were progressively observed throughout the 0.5–3.0 ppm region, and their integrated intensities increased proportionally with backbone length [40, 41]. These results confirmed that the SMI series was successfully synthesized with the intended molecular structures. Moreover, no additional peaks attributable to impurities or unreacted species were detected, indicating the high chemical purity of the SMIs used in this study.

The thermal stability and suitability of the synthesized SMIs for vapor‐phase processing were evaluated by thermogravimetric analysis (TGA), and the results are presented in Figure 2b and Figure S2. As shown in Figure S2, all SMIs exhibited a single mass‐loss behavior, with complete mass loss observed at temperatures above approximately 250°C. The onset temperature of evaporation (*T*_o__n__s__e__t_), defined as the temperature at which mass loss begins, is shown in Figure 2b. The method used to determine *T*_o__n__s__e__t_, based on the intersection of the baseline and the tangent drawn at the steepest slope of the mass‐loss curve is provided in Figure S2. A gradual increase in *T*_o__n__s__e__t_ was observed with increasing alkyl backbone length. Specifically, *T*_o__n__s__e__t_ increased from approximately 153.6°C for TMPMeS to approximately 202.3°C for TMPHxS. This trend can be attributed to the increased molecular weight and enhanced intermolecular van der Waals interactions associated with the longer carbon backbones [42, 43]. Notably, all SMIs in the *n* = 1–6 range exhibited onset temperature of evaporation above the AS‐ALD process temperature of 150°C employed in this study.

Considering the short exposure time in each ALD cycle, this suggests that significant thermal degradation is limited under the applied process conditions. Furthermore, to evaluate the stability of the adsorbed SMIs, water contact angle (WCA) measurements were performed after SMI exposure at different temperatures (100°C–300°C), as shown in Figure S3. The WCA values remained largely unchanged up to 200°C, indicating that the adsorbed SMI layer is preserved within the typical ALD process temperature range (150°C–250°C). Although a gradual change in WCA was observed after annealing at 300°C for extended duration, the magnitude of change was not significant, suggesting that no substantial decomposition occurs within this temperature range. Taken together, these results indicate that the SMIs can be applied under commonly used ALD temperature conditions and retain their chemical functionality under vapor exposure and repeated process cycling at 150°C.

Before quantitatively analyzing the surface‐adsorption behavior of the SMIs, we first examined the types of surfaces that selectively interacted with the newly synthesized SMIs. Table S1 compares the WCAs before and after SMI treatment on representative oxide (SiO_2_ and Al_2_O_3_), nitride (Si_3_N_4_ and TiN), and metal (Cu, Pt, and Ru) surfaces. After the SMI treatment, pronounced increases in the WCA of approximately 32.5° and 16.8° were observed on the SiO_2_ and Al_2_O_3_ surfaces, respectively, whereas only minor changes of less than 5° were detected on the nitride and metal surfaces. This substrate selectivity arises from the methoxy‐based alkoxysilane head group, which preferentially reacts with hydroxylated oxide surfaces via condensation reactions to form stable M─O─Si bonds, while such interactions are largely suppressed on nitride and metal surfaces with limited surface –OH groups. The comparable WCA increase observed on both SiO_2_ and Al_2_O_3_ indicates that this interaction is not limited to a specific oxide, but is generally applicable to hydroxylated oxide substrates, consistent with previous reports on alkoxysilane‐based surface modification across various oxide materials [44, 45]. Based on this general oxide affinity and the clear contrast with non‐oxide surfaces, SiO_2_ was selected as a representative model substrate for subsequent systematic analysis.

**FIGURE 3:**
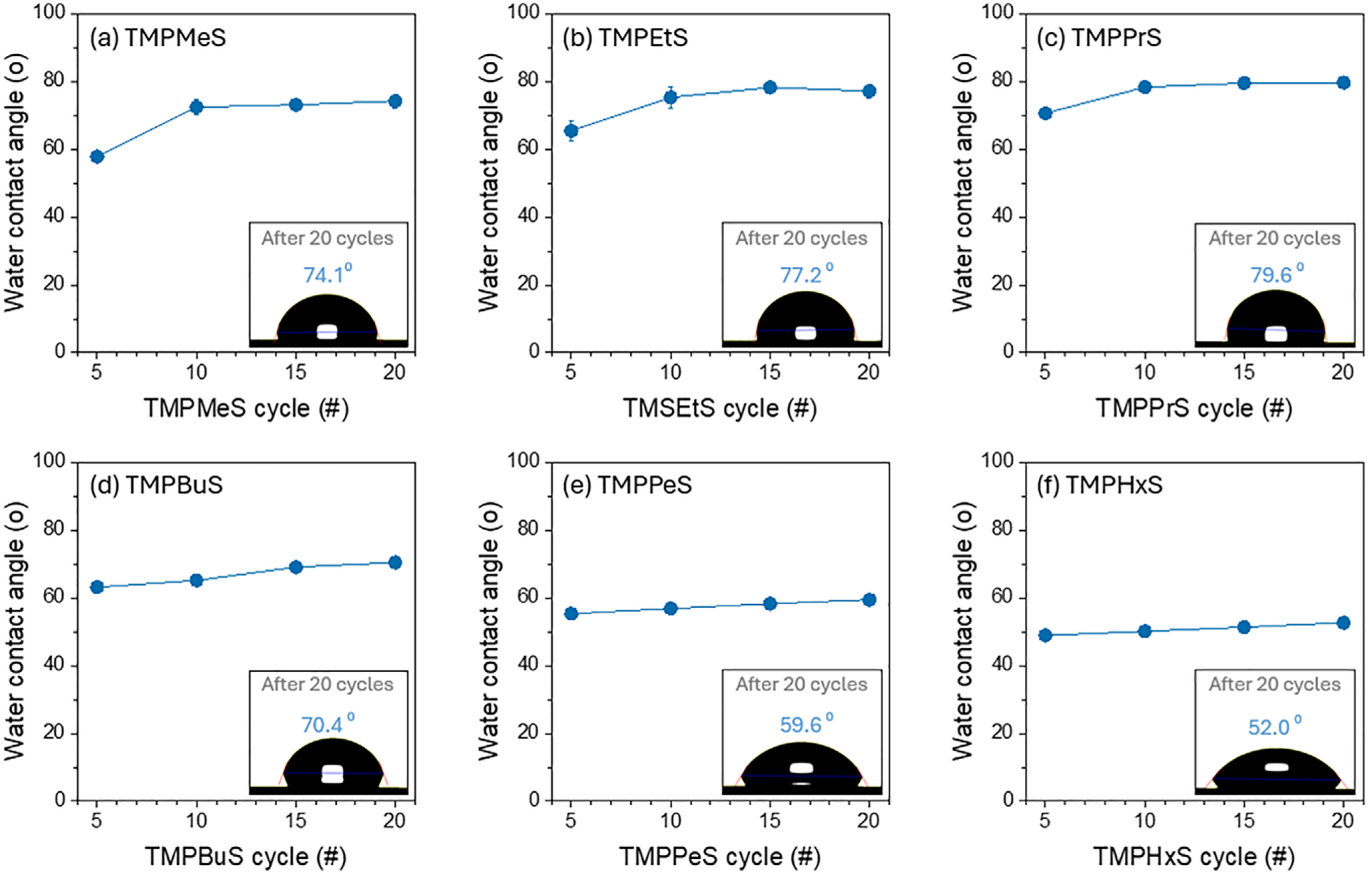
Evolution of WCA on SiO_2_ surfaces as a function of SMI dosing cycles for (a) TMPMeS, (b) TMPEtS, (c) TMPPrS, (d) TMPBuS, (e) TMPPeS, and (f) TMPHxS. Inset figures show optical images of water droplets after 20 SMI dosing cycles with the corresponding WCA values.

Figure 3 shows the evolution of the WCA on SiO_2_ surfaces as a function of the number of SMI dosing cycles for SMIs with different carbon‐backbone lengths. Here, a low WCA corresponds to a hydrophilic surface, while a high WCA indicates a hydrophobic surface. Since the pristine SiO_2_ surface is inherently hydrophilic due to surface hydroxyl (–OH) groups, adsorption of SMIs introduces hydrophobic alkylphenyl tail groups, leading to an increase in WCA. The injection, isolation, and purge times were set as 1, 3, and 120 s for each SMI cycle test, respectively. For all SMIs, the WCA showed a gradual rise with increasing dosing cycles and eventually approached saturation, indicating progressive hydrophobic passivation of the surface. However, the magnitude and cycle dependence of the WCA evolution strongly depended on the carbon‐backbone length [13, 39]. For SMIs with relatively short backbones, namely TMPMeS, TMPEtS, and TMPPrS (Figure 3a–c), relatively low WCA values were observed during the initial five cycles. With an increasing number of cycles, the WCA increased rapidly and then showed a noticeably slower increase after approximately 10–15 cycles. In addition, as the backbone length increased from methyl to propyl, the WCA values in the high‐cycle region (after 20 cycles) progressively increased to 74.1°, 77.2°, and 79.6°, for TMPMeS, TMPEtS, and TMPPrS, respectively. In contrast, SMIs with longer backbones, including TMPBuS, TMPPeS, and TMPHxS (Figure 3d–f), exhibited a more gradual and nearly linear increase in the WCA with increasing cycle number. Notably, the WCA values beyond 20 cycles did not increase further with backbone length and instead showed a decreasing trend. In particular, TMPPeS and TMPHxS yielded WCA values below 60°, indicating relatively weak surface hydrophobization despite repeated dosing.

The results in Figure 3 demonstrate that the SMI adsorption behavior on SiO_2_ does not vary monotonically with the carbon‐backbone length. Instead, WCA evolution can be classified into two distinct regimes depending on the backbone length, characterized by differences in the cycle dependence, growth rate, and high‐cycle behavior. These backbone‐length‐dependent differences in adsorption behavior are discussed in greater detail through subsequent computational analyses.

**FIGURE 4:**
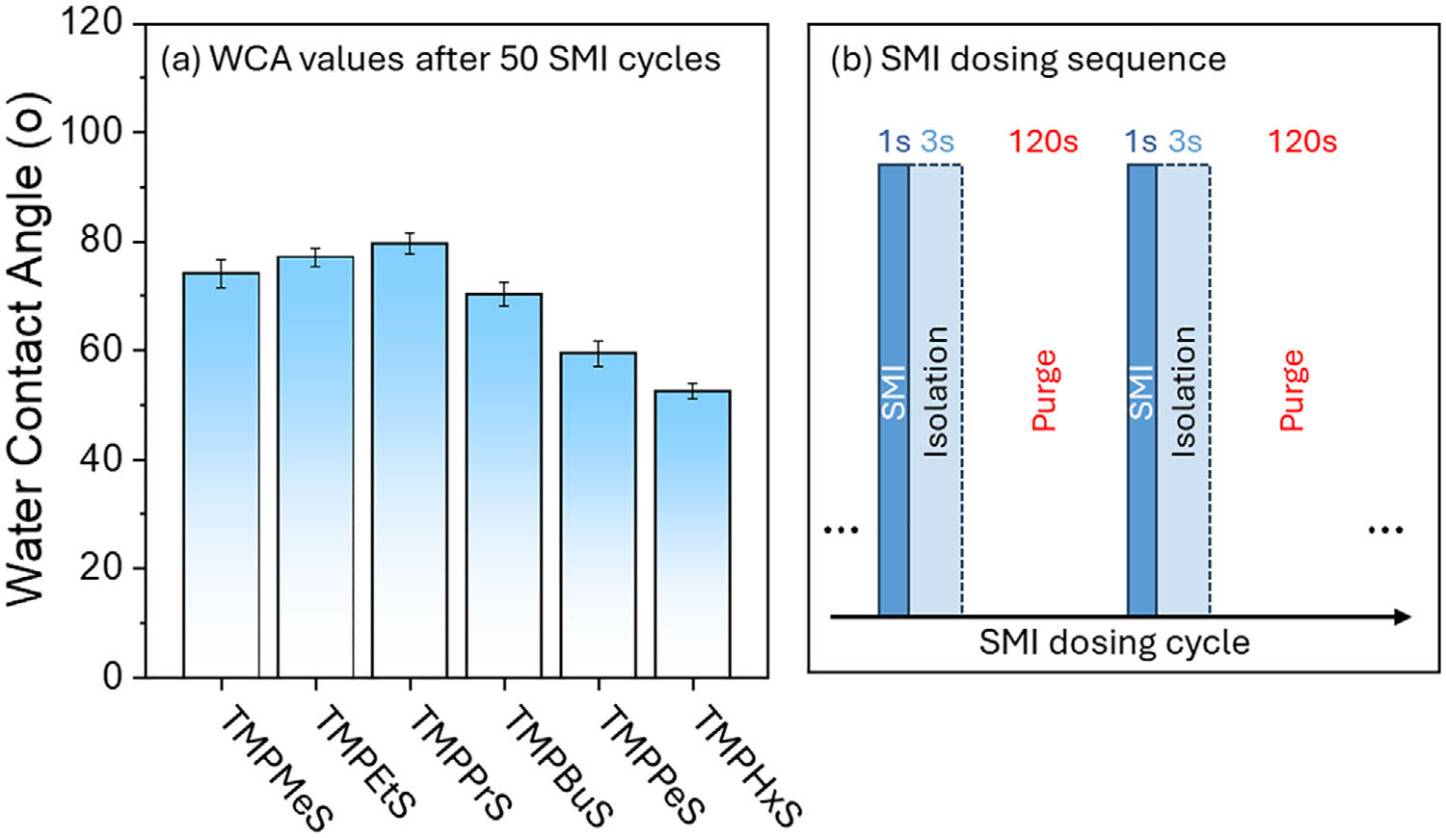
Surface adsorption behavior of SMIs under saturated dosing conditions. (a) WCA values after 50 dosing cycles as a function of carbon backbone length. (b) Schematic of the SMI dosing sequence (1 s injection – 3 s isolation – 120 s purge per cycle).

Under saturated dosing conditions, SMIs exhibit distinct adsorption behavior, as summarized in Figure 4. Based on the WCA evolution in Figure 3, which reaches a saturation after approximately 20 cycles, a dosing condition of 50 cycles was selected to ensure sufficient surface coverage. Under these conditions, the measured WCA serves as a descriptor of the resulting adsorption configurations. As shown in Figure 4a, the WCA values after 50 dosing cycles exhibit a clear dependence on the carbon backbone length, reaching a maximum at an intermediate backbone (TMPPrS) and decreasing for longer chains. This trend indicates the presence of an optimal backbone length for effective surface passivation and suggests that the adsorption behavior is governed by the stability and configuration of the adsorbed molecules rather than their total coverage. These results were obtained under the dosing sequence shown in Figure 4b (*1 s* injection, *3 s* isolation, and *120 s* purge per cycle), where sufficiently long purge times remove weakly bound species. Accordingly, the measured WCA reflects intrinsically stable adsorption configurations rather than accumulation‐driven effects. Thermal annealing results (Figure S4) further support this interpretation. Although the WCA gradually decreases with increasing annealing temperature, the backbone‐length‐dependent trend is preserved. The absence of a sharp WCA drop indicates that most adsorbed species remain thermally stable, confirming that the observed behavior originates from robust adsorption states. Overall, these results demonstrate that the adsorption behavior of SMIs is governed by molecular structure, and that the peak‐and‐decline behavior with increasing backbone length cannot be explained solely by surface coverage. To elucidate the origin of this structure‐dependent behavior, further insights into adsorption configurations and stability are obtained through subsequent simulation analysis.

**FIGURE 5:**
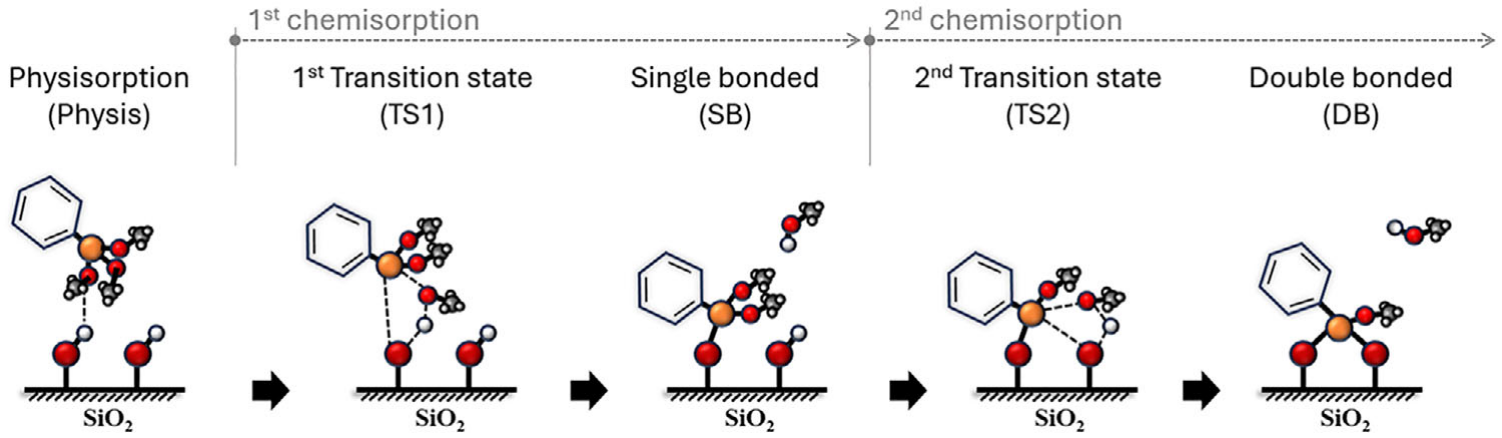
Schematic of the stepwise adsorption process of an SMI molecule on the SiO_2_ surface. The adsorption proceeds through an initial physisorption step, followed by two sequential chemisorption steps, represented by physisorption (Physis), the first transition state (TS1), the single‐bonded state (SB), the second transition state (TS2), and the double‐bonded state (DB).

Figure 5 presents a schematic of the adsorption pathway of an SMI molecule on the surface from the initial physisorbed (Physis) state to the final chemisorbed configuration in a stepwise manner [21]. The SMI molecule employed in this study has an alkoxysilane head group bearing three methoxy ligands; in the final state, two of these ligands are expected to react with the surface to predominantly form a double Si─O─Si bonded (DB) configuration, as commonly reported for alkoxysilane adsorption on hydroxylated oxide surfaces. The adsorption process begins when an SMI molecule approaches the surface hydroxyl groups through weak van der Waals interactions, forming a Physis state. In this state, the SMI molecule undergoes a condensation reaction between a methoxy ligand in the head group and surface hydroxyl group, leading to the formation of the first Si─O─Si bond. This process proceeded through the first transition state (TS1), resulting in a single‐bond (SB) configuration. This reaction can be understood as a typical alkoxysilane‐condensation reaction, in which the surface –OH group acts as a nucleophile, attacking the Si–OCH_3_ bond, accompanied by the release of methanol (CH_3_OH).

Subsequently, a second chemisorption step occurs via an additional condensation reaction with a neighboring surface hydroxyl group. This step passes through the second transition state (TS2) and leads to a DB configuration in which the SMI molecule is strongly anchored to the surface via two covalent Si─O─Si bonds. While mono‐dentate configurations may form at early stages due to minimal geometric constraints, they are generally less stable and more susceptible to desorption. In contrast, bi‐dentate bonding provides enhanced stability while remaining geometrically accessible, and is therefore typically favored. Although one reactive methoxy ligand remains in the head group, the formation of a third Si─O─Si bond is expected to be limited under practical surface conditions. This is because the molecular degrees of freedom become significantly restricted after two bonds are formed, and the spatial distribution of surface hydroxyl groups, along with steric constraints of the molecular structure, hinders further bond formation [46, 47]. Moreover, the DB configuration provides sufficient stabilization energy; thus, the thermodynamic driving force is unlikely to compensate for the additional structural distortion required for a third bond. Consequently, the final accessible adsorption structure is the DB configuration. While tri‐dentate configurations cannot be completely ruled out, they are expected to be minor under typical conditions. Accordingly, the DB configuration is considered the predominant adsorption structure rather than an exclusive one.

Thus, the adsorption of SMI does not proceed in a single step, but follows a multistep reaction pathway comprising an initial Physis state followed by two successive chemisorption steps, each involving intermediate states with different bonding strengths and structural flexibilities. This adsorption pathway provides a microscopic basis for understanding the dependence of the adsorption behaviors observed in Figures 3 and 4 on the purging conditions and molecular structure, particularly in terms of the relative stability of different adsorption configurations.

**FIGURE 6:**
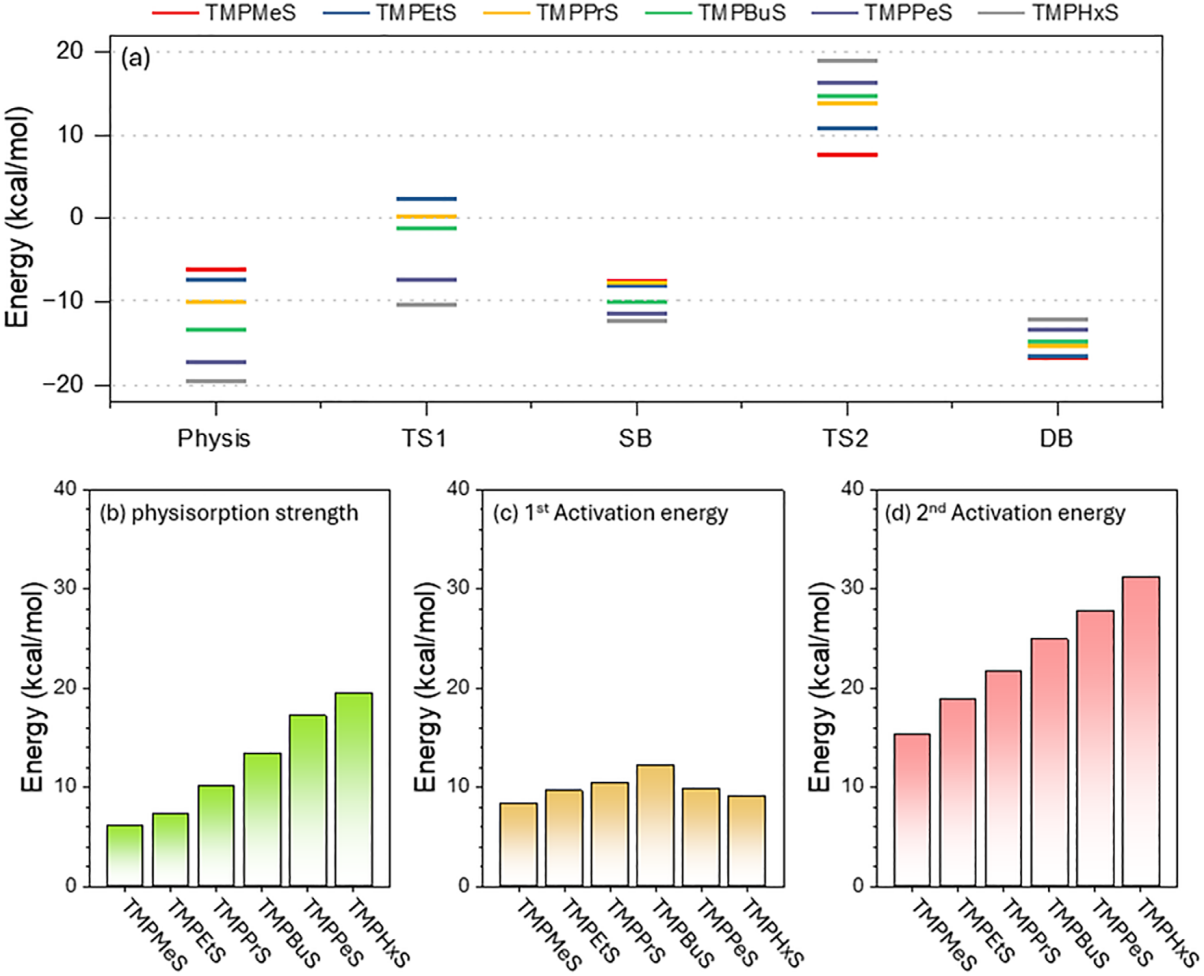
(a) Calculated adsorption energies of trimethoxysilane‐based SMIs with different alkyl backbone lengths at each adsorption state (Physis, TS1, SB, TS2, and DB). (b) Physisorption strength of the SMIs. (c) First activation energy corresponding to the transition from physisorption to the SB state. (d) Second activation energy corresponding to the transition toward the DB state.

Based on the adsorption pathway illustrated in Figure 5, the stepwise energies of Physis, TS1, SB, TS2, and DB were calculated using DFT and are summarized in Figure 6a, with the numerical values listed in Table S2. Figure 6b shows that the physisorption strength progressively increased with increasing backbone length. Here, the physisorption energies correspond to the adsorption of the first SMI molecule on a hydroxylated surface. This trend reflects the enhancement of the dispersion interactions and enlarged contact area between the molecule and surface as the length of the alkyl chain increases. As the backbone and phenyl tail grow, the van der Waals interaction footprint increases, stabilizing the Physis state, even in the absence of covalent‐bond formation [48].

Figure 6c,d shows the activation energies associated with the two‐step chemisorption process. As shown in Figure 6c, TS1 did not follow a simple linear or proportional dependence on the backbone length. Instead, TS1 initially increased from TMPMeS to TMPBuS and then slightly decreased for TMPPeS and TMPHxS. This nonlinear behavior indicates a competition between steric hindrance and conformational efficiency. As the backbone length increased, steric congestion near the surface increased the energetic cost of reorganizing the molecule to form the first Si─O─Si bond. However, for longer chains, the increased conformational flexibility and stronger stabilization of the Physis state allowed the molecule to adopt bent or tilted configurations, partially accommodating this penalty and effectively lowering the relative barrier for the first chemisorption step.

In contrast, a markedly different trend is observed for the second activation barrier (TS2), as shown in Figure 6d. The TS2 barrier increased steeply with increasing backbone length. Once the first Si─O─Si bond was formed, the molecule was anchored to the surface, and the formation of the second bond required the substantial rearrangement and distortion of the molecular backbone. As the alkyl chain lengthened, this rearrangement induced increasingly severe steric congestion and intramolecular strain, making the second chemisorption step progressively less favorable [49, 50]. This structural origin was further supported by the DFT‐optimized adsorption configurations shown in Figure S5. For TMPHxS, two representative geometries were considered: one in which the alkyl backbone extended away from the surface (Case 1) and another in which the backbone was closer to the surface (Case 2). The Case 1 configuration was significantly more stable (−12.1 kcal/mol) than that of Case 2 (−6.3 kcal/mol), indicating that long‐backbone SMIs preferentially adopt an upright or lifted posture rather than maximizing the surface contact of the backbone. Although this geometry stabilized the Physis and SB states, it made the formation of a second Si─O─Si bond geometrically more demanding, thereby contributing to the steep increase in the TS2 barrier with increasing backbone length.

Consequently, although long‐backbone SMIs are strongly stabilized in the Physis state, the formation of the DB chemisorbed configuration was kinetically suppressed because of the rapidly increasing TS2 barrier. This energetic tradeoff explains why long‐backbone SMIs tend to remain in weakly or singly bonded states and are relatively easily removed under long‐purge conditions, as observed for TMPPeS and TMPHxS. However, short‐backbone SMIs such as TMPMeS and TMPEtS can proceed to form stable DB chemisorption structures. Consequently, the adsorption geometry became increasingly anisotropic and extended as the backbone length increased. This is directly visualized in the representative adsorption footprints shown in Figure S6, where the long‐backbone SMIs exhibited significantly larger and more elongated surface projections than their short‐backbone counterparts.

The DFT calculations described above elucidated the adsorption energetics and bonding stability of individual SMI molecules; however, further analysis is required to understand how these behaviors translate into collective packing and surface coverage. Therefore, RSA simulations were performed based on the single‐molecule adsorption footprints derived from Figure 6 to quantitatively evaluate the influence of the molecular geometry on the collective surface packing behavior. DFT captures the energetics and stability of individual adsorption configurations, whereas the RSA describes how the molecular shape and footprint statistically limit the achievable surface coverage under steric exclusion. The 2D adsorption footprints of each SMI were constructed from the molecular geometries shown in Figure S7a–f. The molecular dimensions were measured using Schrödinger software, and each molecule was approximated as an ellipse defined by its major and minor axes. The resulting elliptical 2D footprints and their dimensions are summarized in Table S3 and were used as input objects for the MATLAB‐based RSA simulations. All simulations were performed on a 300 Å × 300 Å substrate, where footprints were randomly and sequentially placed without overlap until no further insertion was possible. A strict boundary condition was imposed to prohibit adsorption events that crossed the substrate boundary.

**FIGURE 7:**
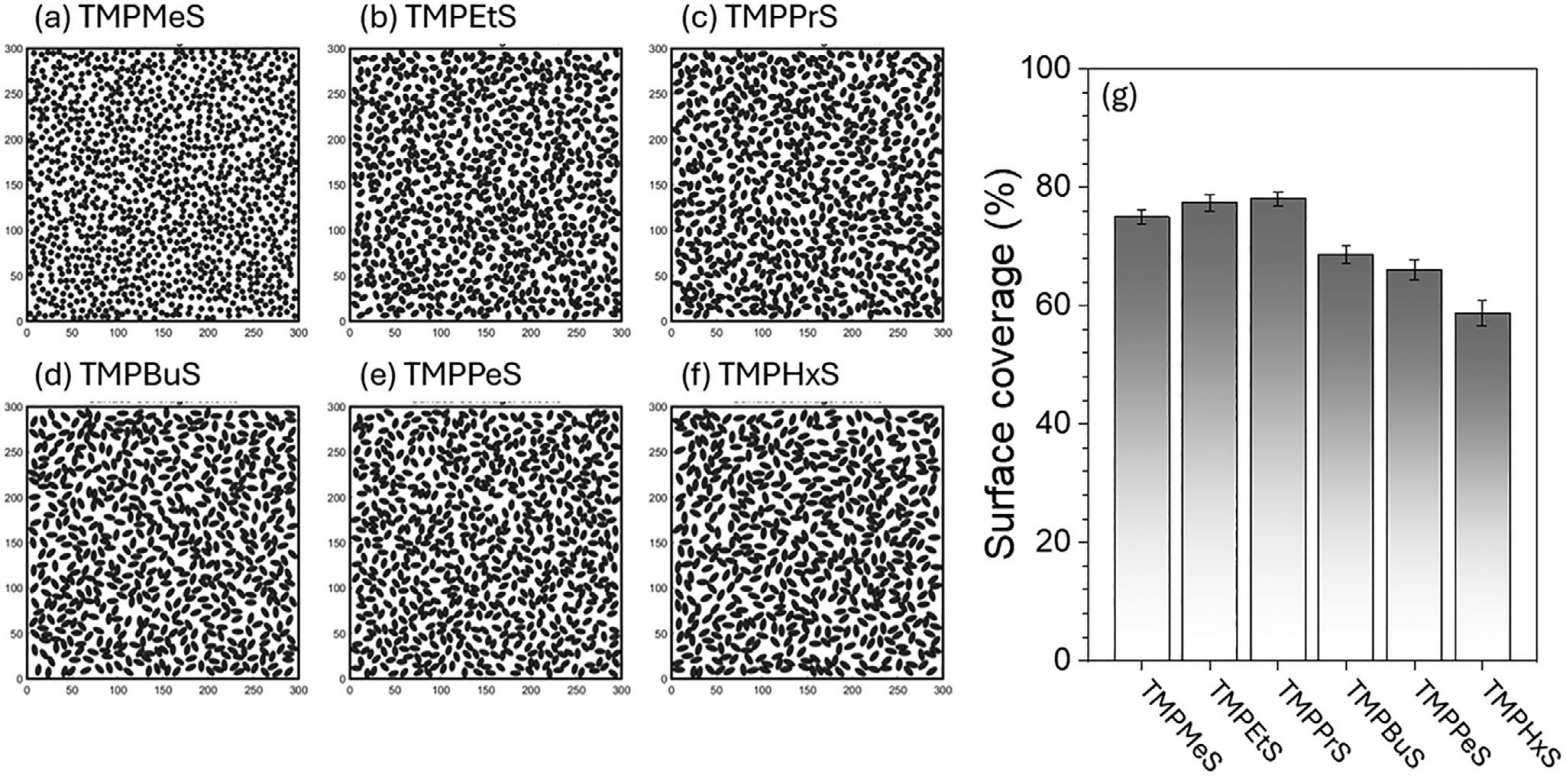
(a–f) Final adsorption configurations of trimethoxysilane‐based SMIs (TMPMeS, TMPEtS, TMPPrS, TMPBuS, TMPPeS, and TMPHxS) obtained from RSA calculations. (g) Surface coverage (%) calculated from the RSA results for each SMI.

The representative final packing configurations obtained from the RSA simulations for each SMI are shown in Figure 7a–f, and the corresponding surface coverages are summarized in Figure 7g. For each SMI, the reported coverage value represents the average of ten independent RSA runs, and the configurations shown in Figure 7a–f correspond to the runs with the smallest deviation from the mean value. The RSA results clearly show that the achievable surface coverage does not increase monotonically with molecular size or carbon‐backbone length. As shown in Figure S8a, smaller SMIs could occupy the surface in larger numbers, whereas larger SMIs could accommodate fewer molecules because of their larger excluded areas per molecule. However, as shown in Figure S8b, the footprint area of an individual molecule increased with increasing backbone length, which partially compensated for the decrease in the number of adsorbed molecules. Consequently, the total surface coverage was determined by the tradeoff between the number of adsorbed molecules and area blocked by each molecule. As a result of this balance, the surface coverage exhibited a nonmonotonic dependence on the backbone length, as shown in Figure 7g. It reached its maximum at an intermediate backbone length, rather than increasing proportionally with the molecular size. When the backbone was too short, many molecules could be adsorbed; however, each molecule blocked only a small area, thereby limiting the total coverage. Conversely, when the backbone was too long, each molecule occupied a large area; however, the number of molecules that could be accommodated on the surface was significantly reduced owing to steric exclusion and inefficient packing. An optimal balance between these two competing effects was achieved at an intermediate backbone length, which yielded the highest attainable surface coverage.

**FIGURE 8:**
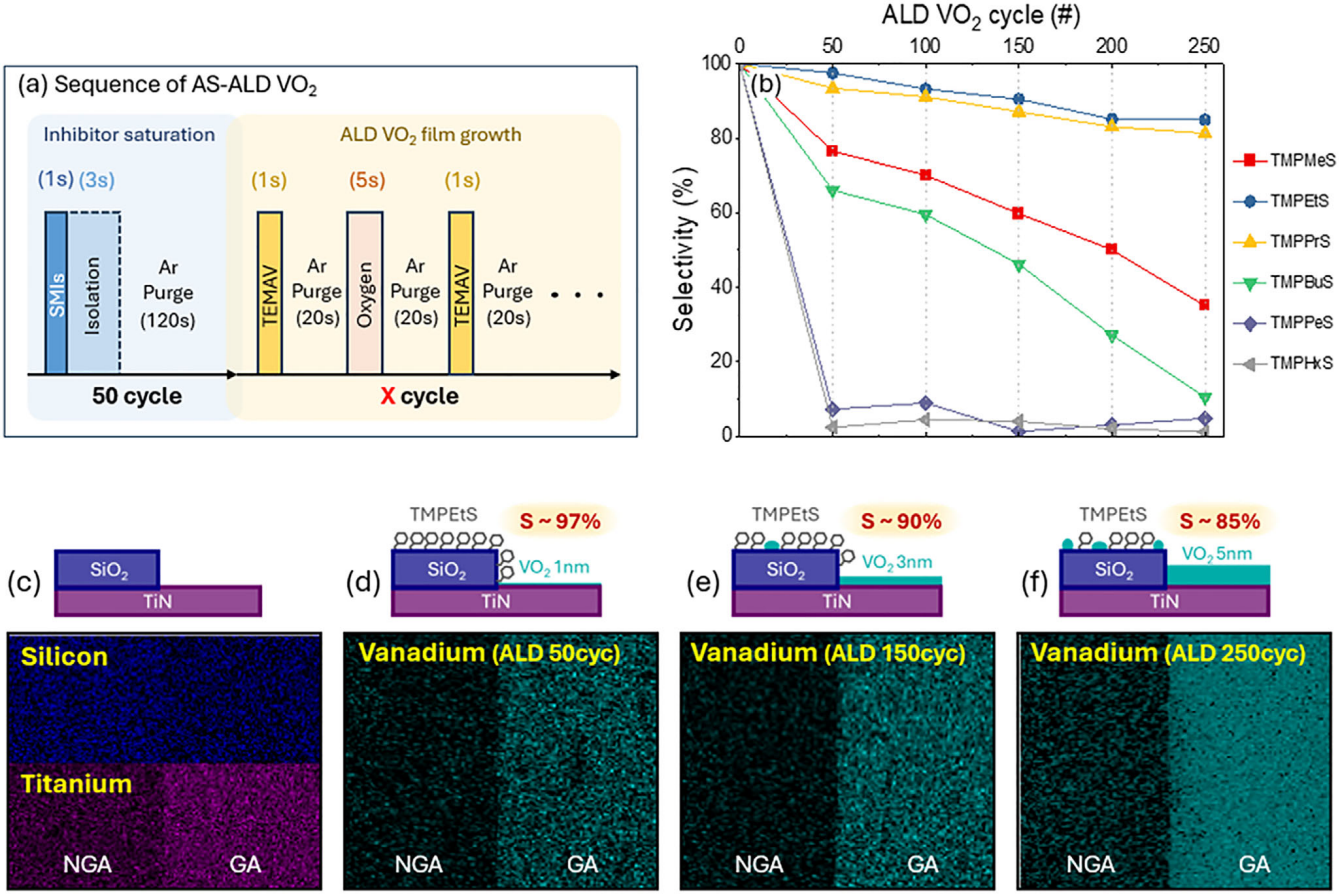
(a) Schematic of the VO_2_ AS‐ALD process sequence, consisting of SMI saturation (50 cycles) followed by VO_2_ ALD cycles. (b) Selectivity of VO_2_ deposition as a function of ALD cycle number for different trimethoxysilane‐based SMIs. (c) Schematic and corresponding SEM–EDS elemental maps of the SiO_2_/TiN patterned substrate prior to VO_2_ deposition, showing the distributions of silicon and titanium. (d–f) Vanadium elemental maps obtained after VO_2_ AS‐ALD using TMPEtS at (d) 50 (∼1 nm), (e) 150 (∼3 nm), and (f) 250 cycles (∼5 nm).

The AS‐ALD of VO_2_ was performed using the developed series of SMIs to validate whether the SMI molecular‐design principles and adsorption mechanisms derived from the preceding DFT and RSA analyses are applicable to a practical AS‐ALD process. Figure 8a schematically illustrates the AS‐ALD sequence used in this study. The entire sequence, including SMI dosing, purge, and subsequent VO_2_ ALD cycles, was carried out in a continuous in situ manner within the same reaction environment. TiN and SiO_2_ were selected as the growth and nongrowth areas, respectively. After SMI dosing, a long purge time of 120 s was applied to ensure that only the strongly bound adsorption species remained on the surface. The SMI dosing was fixed at 50 cycles, which was sufficient to assume the saturated coverage of the SiO_2_ surface. Under these conditions, the SMIs selectively adsorbed only on the SiO_2_ surface, whereas negligible adsorption occurred on the TiN surface, followed by the VO_2_ ALD process. As shown in Figure S9a,b, the VO_2_‐deposition process using tetrakis(ethylmethylamino)vanadium(IV) (TEMAV) and O_2_ exhibited self‐limiting saturation behavior with respect to both the precursor dose and O_2_ exposure, confirming that the film growth proceeded via an ALD mechanism. In addition, as shown in Figure S9c, the deposition temperature of 150°C used in this work was within a stable ALD window.

Figure 8b shows the evolution of selectivity as a function of the VO_2_ ALD cycle number for different SMIs. The VO_2_‐layer thicknesses measured in the growth (TiN) and nongrowth (SiO_2_) areas, together with the selectivity calculations, are summarized in Table S4. The results revealed a clear dependence of the selectivity on the carbon‐backbone length of the SMI. The highest and most stable selectivities (>90%) were observed for TMPEtS and TMPPrS, which have ethyl and propyl backbones (*n* = 2 and 3), respectively, even as the ALD cycle number increased. These SMIs were within the optimal backbone‐length window, where sufficiently stable DB chemisorption structures can be formed, as supported by the DFT results (Figures 5 and 6). Moreover, a high surface coverage was achieved based on the RSA simulations (Figure 7). As the backbone length increased beyond this optimal range (*n* ≥ 4), the selectivity gradually deteriorated with increasing ALD cycles. This degradation reflected the increasing kinetic difficulty in forming stable DB chemisorption configurations, combined with a reduction in the effective surface coverage. For the longest‐backbone SMIs (TMPPeS and TMPHxS, with pentyl and hexyl backbones (*n* = 5 and 6), respectively), almost no selectivity was observed, even at an early stage (∼50 cycles). As discussed in the DFT and RSA analyses, these molecules had difficulty forming stable DB chemisorption structures because of the kinetically suppressed second chemisorption step, and they simultaneously exhibited low surface‐packing efficiency. Consequently, they failed to establish an effective blocking layer on the nongrowth surface, leading to the rapid nucleation of VO_2_.

The selectivity of VO_2_ growth on the SiO_2_/TiN patterned substrate was further examined by scanning electron microscopy‐energy‐dispersive X‐ray spectroscopy (SEM–EDS) using TMPEtS, which exhibited the highest and most stable performance. Figure 8c shows the elemental maps of Si and Ti with a simplified schematic of the SiO_2_/TiN pattern used in this study. The full stack structure of the actual pattern is presented in Figure S10a,b, and only the top two layers (SiO_2_/TiN), which directly participated in the AS‐ALD process, are illustrated here for clarity. Because the TiN layer was deposited on the SiO_2_/Si substrate, a Si signal was detected over the entire area, as shown in Figure S10c. Figure 8d–f shows the evolution of the V elemental distribution after 50, 150, and 250 VO_2_ ALD cycles following TMPEtS adsorption. In the TiN‐growth area, the V signal increased progressively with an increasing number of ALD cycles, whereas in the SiO_2_ nongrowth area, the V signal remained strongly suppressed over the entire cycle range. This clearly shows that the high selectivity observed in Figure 8b was spatially well‐maintained on the patterned substrate.

## 3 Conclusion

In this study, we established a molecular‐level design rule for SMIs in AS‐ALD by correlating the adsorption chemistry, molecular geometry, and macroscopic selectivity performance. By synthesizing a series of trimethoxysilane‐based SMIs with systematically varying carbon‐backbone lengths, we demonstrated that the inhibition performance is governed not by a single factor, but by the coupling of adsorption stability and surface‐packing efficiency. DFT calculations revealed that SMI adsorption on hydroxylated SiO_2_ followed a three‐step process, In this process, increasing the backbone length strengthened physisorption but simultaneously hindered the formation of the most stable chemisorbed state owing to steric constraints. RSA simulations revealed that the achievable surface coverage was governed by a tradeoff between the number of adsorbed molecules and steric exclusion arising from the molecular‐footprint size, leading to a nonmonotonic dependence on the backbone length. Therefore, an optimal intermediate backbone length that balanced the adsorption stability and surface‐packing efficiency emerged. These theoretical insights were directly validated by VO_2_ AS‐ALD experiments, where only SMIs with intermediate backbone lengths achieved both strong chemisorption stability and high surface coverage, resulting in consistently high selectivity (>90%). In contrast, SMIs with long backbones exhibited reduced selectivity owing to their insufficient adsorption stability or poor surface‐blocking efficiency. Overall, this study demonstrates that effective AS‐ALD inhibitors must simultaneously satisfy adsorption stability and packing efficiency requirements, which are governed by the backbone length. This defined a clear molecular‐size range for optimal inhibitor performance and provided a general design principle for rational inhibitor engineering in AS‐ALD.

## 4 Experimental Section

### 4.1 Materials and SMI Synthesis

#### 4.1.1 Materials

(2‐Bromoethyl)benzene, 1,5‐dibromopentane, and 3‐phenylpropyl bromide were purchased from Tokyo Chemical Industry (Japan). Benzyl bromide, 1,4‐dibromobutane, and 1,6‐dibromohexane were purchased from Thermo Fisher Scientific (USA). Tetramethyl orthosilicate, bromobenzene, and n‐butyllithium were purchased from Sigma–Aldrich (USA). All the solvents were dried over activated molecular sieves before use. All glassware was dried in an oven at 80°C to remove residual moisture. All synthetic procedures were performed under an argon atmosphere. ^1^H NMR spectra were recorded on a spectrometer (AVANCE III HD 400, Bruker, Germany). Chemical shifts (ppm) were calibrated using CDCl_3_.

#### 4.1.2 Synthesis of Trimethoxysilane‐Based SMIs

The starting materials, a series of 1‐bromo‐n‐phenylalkanes (*n* = 4, 5, and 6, corresponding to alkane = butane, pentane, and hexane, respectively), were prepared using the following procedure. n‐butyllithium (4.8 mL, 2.5 m in hexane) was added dropwise to a stirred solution of bromobenzene (10 mmol) in anhydrous diethyl ether (50 mL) at −78°C. The reaction mixture was stirred at ‐78°C for 1 h, and then warmed to 0°C. A solution of dibromoalkane (15 mmol) in anhydrous diethylether (10 mL) was added to the reaction mixture with stirring. The reaction solution was stirred at 0°C for 6 h. After the completion of the reaction, the solvent was evaporated under reduced pressure. The crude product was dissolved in hexane (30 mL) and washed three times with saturated brine (30 mL  ×  3). The organic phase was dried over anhydrous MgSO_4_ and filtered, and the filtrate was concentrated under reduced pressure. The resulting product was purified via distillation.

1‐Bromo‐n‐phenylalkane (*n* = 1–6, corresponding to alkane = methane, ethane, propane, butane, pentane, and hexane, respectively) (10 mmol) was added to dried Mg turnings (12 mmol) in anhydrous diethyl ether (30 mL) under reflux. The reaction mixture was stirred for 1 h. The resulting Grignard reagent was then added dropwise to the solution of tetramethyl orthosilicate (11 mmol) in anhydrous diethylether (10 mL) at 0°C under stirring. The reaction mixture was stirred overnight. After the completion of the reaction, the mixture was washed three times with saturated brine. The organic phase was dried over anhydrous MgSO_4_ and filtered, and the filtrate was concentrated under reduced pressure. The resulting product was purified via distillation.

### 4.2 Substrate Preparation and AS‐ALD Process

Four types of oxide and nitride substrates (SiO_2_, Al_2_O_3_, Si_3_N_4_, and TiN) were prepared via ALD, and Cu, Pt, and Ru films were deposited via sputtering. Prior to the experiments, all substrates were cleaned sequentially in acetone, isopropyl alcohol, and deionized water to remove possible organic and particulate contaminants. All the deposition and surface‐treatment experiments were conducted within one week of the substrate preparation, and the samples were stored in a nitrogen‐filled glovebox during this period to minimize ambient contamination and surface oxidation. Before SMIs adsorption, the SiO_2_ and Al_2_O_3_ surfaces were additionally treated with ultraviolet–ozone exposure prior to SMI dosing to ensure a sufficiently hydroxylated surface. In contrast, the Si_3_N_4_ amd TiN substrates were dipped in a 100:1 diluted HF solution for 10 s to remove the native oxide layer and immediately transferred to the reactor for subsequent experiments. For the AS‐ALD experiments, SiO_2_ and TiN substrates were used as the nongrowth and growth surfaces, respectively. All SMI dosing and VO_2_ ALD processes were carried out in situ in the same reaction chamber at a substrate temperature of 150°C. The reactor used in this study was a hot‐wall, lateral‐flow ALD reactor (NexusBe Ltd. Co., Republic of Korea).

Six trimethoxysilane‐based SMIs were used in this study: Trimethoxyphenyl(methyl)silane (TMPMeS), Trimethoxyphenyl(ethyl)silane (TMPEtS), trimethoxyphenyl(propyl)silane (TMPPrS), trimethoxyphenyl(butyl)silane (TMPBuS), trimethoxyphenyl(pentyl)silane (TMPPeS), and trimethoxyphenyl(hexyl)silane (TMPHxS). All SMIs were liquid at room temperature. To ensure sufficient vapor pressure and stable delivery into the reactor, the SMI canisters were heated as follows: TMPMeS and TMPEtS to 80°C; TMPPrS, TMPBuS, and TMPPeS to 90°C; and TMPHxS to 95°C. An Ar‐assisted dosing method was employed for SMI delivery: the canister was pressurized to approximately 1–2 Torr using Ar (500 sccm), and vapor was introduced into the chamber. Ar (500 sccm) was used during purging. The chamber pressure during the SMI dosing was maintained at 300 mTorr.

VO_2_ ALD was performed in the same chamber at the same substrate temperature (150°C) immediately after SMI dosing without breaking the vacuum. TEMAV (SK Trichem, Republic of Korea) was used as the vanadium precursor, and high‐purity O_2_ (99.999%) was used as the oxidant. The TEMAV precursor was contained in a stainless‐steel canister and maintained at 50°C during the process. Similar to the SMI dosing, an Ar‐assisted delivery method was used, in which the canister was pressurized to approximately 1–2 Torr using Ar (500 sccm) to ensure a stable precursor supply. The O_2_ flow rate was 50 sccm, and Ar (500 sccm) was used as both the carrier and purge gas. The chamber pressure during VO_2_ ALD was maintained at 500 mTorr.

### 4.3 Material and Surface‐Property Analysis

^1^H NMR measurements were performed using a spectrometer (AVANCE III HD, Bruker, Germany) operating at 400 MHz, and all chemical shifts were calibrated against the residual proton resonance of CDCl_3_. TGA was conducted using an analyzer (TGA 2, Mettler Toledo, Switzerland) under a nitrogen atmosphere, with the temperature increased from 30°C to 500°C at a rate of 10°C min^−1^. WCA measurements were performed using a contact‐angle analyzer (Phoenix P300, S.E.O. Ltd., Republic of Korea) to evaluate the surface wettability and energy characteristics of the SMI‐treated substrates. Deionized water was used as the probe liquid to assess the polar‐surface properties. For each SMI condition, 10 samples were measured, and the reported WCA values were obtained by averaging the left and right contact angles of each sample. The contact angles were calculated using Surfaceware 8 software (ver. 10.11) provided by the manufacturer. X‐ray fluorescence (XRF) analysis was performed using a spectrometer (ARL QUANT'X EDXRF, Thermo Fisher Scientific, USA)) to quantitatively analyze the elemental composition of the VO_2_ films deposited by AS‐ALD. The instrument operated in the energy‐dispersive XRF mode using a Rh anode X‐ray tube as the excitation source. The beam diameter was set to 8.8 mm to ensure a sufficient spatial resolution for area‐selective measurements. To improve sensitivity and quantitative accuracy, all measurements were conducted under vacuum conditions, which minimized background interference, particularly for light elements. Each spectrum was acquired with an integration time of 10 s per scan and the final spectra were obtained by averaging more than 10 scans to enhance the signal‐to‐noise ratio. The quantification of V, Si, N, O, and Ti was performed using a fundamental parameter calibration model incorporating the Compton‐scattering correction. The selectivity of the VO_2_ deposition was evaluated by converting the XRF intensities measured in the growth (TiN) and nongrowth (SiO_2_) areas into thickness values and comparing them. SEM–EDS was performed using a nanoprobe (PHI 700Xi, ULVAC‐PHI, Japan) equipped with a field‐emission electron source. The SEM beam conditions were set as follows: at an accelerating voltage of 20 kV and a beam current of 1 nA, the probe size was < 7 nm; at 10 kV and 1 nA, the probe size was < 17 nm; and at 10 kV and 10 nA, the probe size was < 22 nm.

### 4.4 Simulation and Computational Methods

DFT calculations were performed to evaluate the adsorption energetics of SMI molecules on SiO_2_ and TiN surfaces using Maestro Materials Science 2025‐1 (Schrödinger Inc.) on a local workstation equipped with 40 CPU cores, with an average iteration time of approximately 1 ms. The electronic‐structure calculations were carried out using Quantum ESPRESSO. The Perdew–Burke–Ernzerhof functional was employed within the generalized gradient approximation, whereas the B3LYP‐D3 hybrid functional was used for selected calculations to account for dispersion interactions, together with the LAC3P+ norm‐conserving pseudopotential basis set. The substrates were modeled as hydroxyl‐terminated SiO_2_ and TiN slabs. For the SiO_2_ model, all atoms except for the surface hydroxyl groups and topmost Si atoms were fixed during geometry optimization. The calculation models included SiO_2_ (100) and TiN (100) surfaces constructed using a 4 × 4 × 3 supercell with a vacuum layer of 15 Å to prevent spurious interactions between periodic images. The adsorption energies of the SMI molecules were calculated for both the SiO_2_ and TiN surfaces. The convergence criteria were set to 1 × 10^−5^ eV per atom for the total energy and 0.01 eVÅ^−1^ for the atomic forces. Brillouin‐zone sampling was performed using a Γ‐centered Monkhorst–Pack k‐point grid of 3 × 3 × 1. Geometry optimizations were performed to ensure structural stability prior to adsorption‐energy analysis. Transition‐state energies were estimated using the climbing‐image nudged elastic band method, enabling the accurate determination of the reaction‐energy barriers along the adsorption pathways.

RSA simulations were performed using customized MATLAB scripts on a local workstation equipped with 16 CPU cores. The substrate was modeled as a continuous 2D plane with dimensions of 300 × 300 Å^2^ with strict boundary conditions such that the adsorption of molecules crossing the boundary was not allowed. The 2D footprints of the six SMI molecules were constructed by approximating their geometries (obtained from the DFT‐optimized structures) as ellipses defined by their major and minor axes. During the adsorption process, overlap between footprints was strictly prohibited. Each simulation comprised more than 10 000 placement attempts. Following the stochastic RSA algorithm, the molecules were randomly positioned and oriented on the substrate at angular intervals of 15°. Each trial placement was accepted or rejected based on an overlap check. The simulation was terminated when no further molecules were placed, corresponding to the jamming limit. The primary output metrics included the packing density (surface‐coverage percentage) and interparticle‐spacing distribution.

## Conflicts of Interest

The authors declare no conflicts of interest.

## Supporting information




**Supporting File**: advs75705‐sup‐0001‐SuppMat.docx.

## Data Availability

The data that supports the findings of this study are available in the supplementary material of this article.
