# Supplementary material for: Backbone‐Length‐Optimized Inhibitors Deliver Long‐Retention Selectivity in Area‐Selective ALD of VO2
Source: Adv Sci (Weinh). 2026 May 15;13(45):e75705. doi: 10.1002/advs.75705 (PMC13335821; doi:10.1002/advs.75705)
Supplement: Supplementary file 1 [file ADVS-13-e75705-s001.docx]

Supporting Information

Backbone-Length-Optimized Inhibitors Deliver Long-Retention Selectivity in Area-Selective ALD of VO_2_

*Hae Lin Yang^1, ‡^, Eun Chong Cho,^2,‡^, Minchan Kim^1^, Hye In Park^3^, Ga-young Lee^1^, Seunghwan Lee^4^, Beomseok Kim^4^, Changhwa Jung^4^, Youngkwon Kim^2,*^, and Jin-Seong Park^1*,^*

H.L. Yang, M. Kim, G.-Y. Lee, J.-S. Park

Division of Materials Science and Engineering, Hanyang University, 222 Wangsimni-ro Seongdong-gu, Seoul, 04763, Republic of Korea

Email: jsparklime@hanyang.ac.kr

E.C. Cho, Y. Kim

Thin Film Materials Research Center, Korea, Research Institute of Chemical Technology (KRICT), PO Box 107, Yusung, Daejeon 305-600, Korea

Email: ykkim@krict.re.kr

H.I. Park

Department of Semiconductor Engineering, Hanyang University, 222 Wangsimni-ro Seongdong-gu, Seoul, 04763, Republic of Korea

S. Lee, B. Kim, C. Jung

Semiconductor Research and Development Center, Samsung Electronics Company, 1 Samsungjeonja-ro, Hwaseong-si, Gyeonggi-do, 18448, Republic of Korea

*Corresponding Authors: Dr. Youngkwon Kim ([ykkim@krict.re.kr](mailto:ykkim@krict.re.kr)) and Prof. Jin-Seong Park ([jsparklime@hanyang.ac.kr](mailto:jsparklime@hanyang.ac.kr))

^‡^ Authors contribuit this work equeally.

**Figure S1**. ^1^H NMR spectra of trimethoxysilane-based SMIs with different alkyl backbone lengths: TMPMeS, TMPEtS, TMPPrS, TMPBuS, TMPPeS, and TMPHxS.

Figure S2. TGA curves showing weight (%) as a function of temperature for trimethoxysilane-based SMIs with different alkyl backbone lengths. The inset shows the determination of the onset temperature evaporation (*T*ₒₙₛₑₜ) using the tangent intersection method.

Figure S3. WCAs of SiO_2_ surfaces after SMI dosing as a function of process temperature (100, 200, and 300 ℃) for SMIs with different carbon-backbone lengths: (a) TMPMeS, (b) TMPEtS, (c) TMPPrS, (d) TMPBuS, (e) TMPPeS, and (f) TMPHxS.

**Table S1**. WCAs measured on various substrate surfaces before (W/O SMI) and after (W/ SMI) treatment using SMIs with different carbon-backbone lengths.

| **Various**  **Substrate** | **Water contact angle ( ^o^)** | | | | | | |
| --- | --- | --- | --- | --- | --- | --- | --- |
|  | **W/O SMI** | **W/ SMI** | | | | | |
|  |  | **TMPMeS** | **TMPEtS** | **TMPPrS** | **TMPBuS** | **TMPPeS** | **TMPHxS** |
| **SiO_2_** | 45.6 | 74.1 | 77.2 | 78.1 | 70.4 | 59.6 | 52.0 |
| **Al_2_O_3_** | 51.1 | 63.5 | 66.2 | 67.9 | 61.0 | 53.8 | 48.5 |
| **Si_3_N_4_** | 73.0 | 72.5 | 73.1 | 73.9 | 718. | 69.5 | 68.0 |
| **TiN** | 81.6 | 75.2 | 74.5 | 73.6 | 73.0 | 71.8 | 70.5 |
| **Cu** | 110.0 | 108.5 | 107.9 | 107.4 | 106.8 | 105.6 | 104.9 |
| **Pt** | 107.1 | 103.5 | 102.8 | 102.0 | 101.4 | 100.2 | 99.5 |
| **Ru** | 105.6 | 101.5 | 100.9 | 100.3 | 99.8 | 98.7 | 97.69 |

**Figure S4**. Changes in WCA on SiO_2_ surfaces modified with different SMIs after thermal annealing at 400, 500, and 600 ℃ for 1 h. The WCA values before annealing (“bare”) are also shown for comparison.

**Table S2**. Calculated adsorption energies (Physis, TS1, SB, TS2, and DB), activation energies (Ea1 and Ea2), and physisorption energies (physi) for trimethoxysilane-based SMIs obtained from DFT calculations.

|  | TMPMeS | TMPEtS | TMPPrS | TMPBuS | TMPPeS | TMPHxS |
| --- | --- | --- | --- | --- | --- | --- |
| Physis | - 6.1 | - 7.3 | - 10.1 | - 13.4 | - 17.2 | - 19.5 |
| TS1 | 2.3 | 2.3 | 0.3 | - 1.2 | - 7.4 | - 10.4 |
| SB | - 7.6 | - 8.0 | - 7.7 | - 10.1 | - 11.5 | - 12.3 |
| TS2 | 7.7 | 10.9 | 13.9 | 14.8 | 16.3 | 18.9 |
| DB | - 16.7 | - 16.6 | - 15.3 | - 14.8 | - 13.3 | - 12.1 |
| Ea1 | 8.4 | 9.6 | 10.4 | 12.2 | 9.8 | 9.1 |
| Ea2 | 15.3 | 18.9 | 21.6 | 24.9 | 27.8 | 31.2 |
| Physisorption  energy | 6.1 | 7.3 | 10.1 | 13.4 | 17.2 | 19.5 |

**Figure S5**. Representative DFT-optimized adsorption configurations of TMPHxS illustrating two possible adsorption cases for a long alkyl-chain SMI on the SiO₂ surface. (a,b) Case #1 shows a configuration with the alkyl backbone extending away from the surface, whereas (c,d) Case #2 shows an adsorption configuration in which the alkyl backbone is oriented closer to the surface. (a,c) Side-view and (b,d) top-view configurations are presented for each case.

**Figure S6**. DFT-optimized DB adsorption structures of trimethoxysilane-based SMIs with different alkyl backbone lengths on the SiO_2_ surface: (a,g) TMPMeS, (b,h) TMPEtS, (c,i) TMPPrS, (d,j) TMPBuS, (e,k) TMPPeS, and (f,l) TMPHxS. (a–f) Side-view and (g–l) top-view configurations are shown.

**Figure S7**. Optimized molecular geometries of trimethoxysilane-based SMIs with different alkyl backbone lengths: (a) TMPMeS, (b) TMPEtS, (c) TMPPrS, (d) TMPBuS, (e) TMPPeS, and (f) TMPHxS. Top-view configurations are shown, with selected intermolecular distances and geometric parameters indicated.

**Table S3**. Major- and minor-axis lengths of the 2D elliptical footprints used to approximate the DFT-optimized molecular structures for RSA simulations of TMPMeS, TMPEtS, TMPPrS, TMPBuS, TMPPeS, and TMPHxS.

|  | TMPMeS | TMPEtS | TMPPrS | TMPBuS | TMPPeS | TMPHxS |
| --- | --- | --- | --- | --- | --- | --- |
| Major axis (Å) | 9.80 | 6.77 | 10.8 | 12.4 | 11.4 | 13.4 |
| Minor axis (Å) | 5.95 | 5.95 | 6.05 | 5.97 | 5.94 | 6.19 |

**Figure S8**. (a) Number of adsorbed molecules obtained from RSA calculations for different SMIs. (b) Molecular area (2D footprint) used in the RSA calculations.

**Figure S9**. Growth per cycle (Å/cycle) measured as a function of (a) precursor temperature, (b) oxygen dose time, and (c) substrate temperature. Unless otherwise noted, measurements were performed at a substrate temperature of 120 ℃, a precursor temperature of 50 ℃, an oxygen flow rate of 50 sccm, and an oxygen dose time of 1 s.

**Table S4**. VO_2_ thickness (Å) as a function of ALD cycle number measured on TiN and SMI-treated SiO_2_ surfaces using SMIs with different carbon-backbone lengths (TMPMeS–TMPHxS). The results highlight the variation in inhibition efficiency on SiO_2_ depending on the SMI backbone length, while consistent growth is observed on the TiN surface.

| **VO_2_**  **cycle** | **VO_2_ thickness (Å)** | | | | | | |
| --- | --- | --- | --- | --- | --- | --- | --- |
|  | **TiN surface** | **SiO_2_ surface** | | | | | |
|  |  | **TMPMeS** | **TMPEtS** | **TMPPrS** | **TMPBuS** | **TMPPeS** | **TMPHxS** |
| **0** | 0.0 | 0.0 | 0.0 | 0.0 | 0.0 | 0.0 | 0.0 |
| **50** | 15.1 | 2.0 | 0.8 | 0.8 | 3.1 | 13.0 | 14.4 |
| **100** | 32.0 | 5.6 | 1.7 | 1.9 | 8.1 | 26.7 | 29.2 |
| **150** | 51.0 | 12.8 | 2.6 | 3.5 | 18.7 | 49.6 | 47.0 |
| **200** | 71.0 | 23.6 | 5.7 | 6.6 | 40.7 | 66.8 | 68.1 |
| **250** | 84.0 | 40.4 | 6.8 | 8.7 | 67.9 | 76.2 | 82.0 |

**Figure S10.** (a) Top-view schematic of the laterally patterned SiO_2_/TiN substrate, consisting of two 500 µm × 1000 µm regions for SiO_2_ and TiN, respectively. (b) Cross-sectional schematic of the multilayer stack structure used in this study: Si substrate / thermal SiO_2_ (100 nm) / ALD TiN (10 nm) / ALD SiO_2_ (100 nm). (c) Top-view SEM images of the SiO_2_ and TiN regions, showing the surface morphology of each material.
